# An Open Source Based High Content Screening Method for Cell Biology Laboratories Investigating Cell Spreading and Adhesion 

**DOI:** 10.1371/journal.pone.0078212

**Published:** 2013-10-21

**Authors:** Andre Schmandke, Antonio Schmandke, Maurianne A. Pietro, Martin E. Schwab

**Affiliations:** Brain Research Institute, University of Zurich and Department of Health Sciences and Technology, ETH Zurich, Zurich, Switzerland

## Abstract

**Background:**

Adhesion dependent mechanisms are increasingly recognized to be important for a wide range of biological processes, diseases and therapeutics. This has led to a rising demand of pharmaceutical modulators. However, most currently available adhesion assays are time consuming and/or lack sensitivity and reproducibility or depend on specialized and expensive equipment often only available at screening facilities. Thus, rapid and economical high-content screening approaches are urgently needed.

**Results:**

We established a fully open source high-content screening method for identifying modulators of adhesion. We successfully used this method to detect small molecules that are able to influence cell adhesion and cell spreading of Swiss-3T3 fibroblasts in general and/or specifically counteract Nogo-A-Δ20-induced inhibition of adhesion and cell spreading. The tricyclic anti-depressant clomipramine hydrochloride was shown to not only inhibit Nogo-A-Δ20-induced cell spreading inhibition in 3T3 fibroblasts but also to promote growth and counteract neurite outgrowth inhibition in highly purified primary neurons isolated from rat cerebellum.

**Conclusions:**

We have developed and validated a high content screening approach that can be used in any ordinarily equipped cell biology laboratory employing exclusively freely available open-source software in order to find novel modulators of adhesion and cell spreading. The versatility and adjustability of the whole screening method will enable not only centers specialized in high-throughput screens but most importantly also labs not routinely employing screens in their daily work routine to investigate the effects of a wide range of different compounds or siRNAs on adhesion and adhesion-modulating molecules.

## Introduction

Cell adhesion is known to play a major role in a wide number of processes during development and adulthood, ranging from tissue formation and homeostasis up to regenerative events such as wound closure and inflammatory cell infiltration after injury. Likewise a growing number of diseases such as cancer or chronic inflammation but also of therapeutic interventions such as stem cell transplantations has been identified to rely on adhesion-based events such as migration.

Even though cell-substrate adhesion modulating proteins are classically described to be important for cell migration it becomes increasingly apparent that these molecules can have a wide range of additional functions [1-3]. Vice versa, numerous proteins identified earlier as being involved in adhesion- or migration-unrelated cellular events are increasingly being recognized to also modulate cell attachment, spreading or migratory behavior of cells [4-6]. This principle is nicely demonstrated by the membrane protein Nogo-A which – next to its well established role as a neurite outgrowth inhibitor and repressor of synaptic plasticity [7] – plays a crucial role for adhesion, cell motility and migration *in vitro* as well as *in vivo*. While an increase in motility and migration was detected upon treatment of cells with anti-Nogo-A antibodies as well as in cells from Nogo-A null mice [8], Nogo-A was also shown to promote tangential migration of early-born interneurons from the medial ganglionic eminence [9] and to support neuroblast progression along the rostral migratory stream [10]. Just recently the functional Δ20-domain of Nogo-A was also demonstrated to inhibit cell spreading, adhesion and migration of mouse vascular endothelial cells *in vitro* [11]. Furthermore Nogo-A was hypothesized to play a role in cerebellar granule cell migration during early postnatal layering of the cerebellar cortex [12].

The importance of adhesion dependent mechanisms in biological processes, diseases and for therapeutics has led to a rising demand of pharmaceutical modulators. However, adhesion is complex; the protein interaction network enabling cell – substrate interactions via integrins and the actin cytoskeleton has been suggested to comprise 180 potential signaling nodes [13]. In order to detect compounds able to modulate such a complex network, high throughput methods are essential. However, high-throughput screening facilities are not always available to laboratories and are often rather expensive.

We developed a high content screening approach that can be used in any cell biology laboratory possessing a fluorescent microscope equipped with a fast, automated sampling table to find novel modulators of adhesion and cell spreading. The method is based exclusively on freely available open-source software. We utilized this approach to screen a library of 1040 small compounds, most of which are admitted for neurological indications (NINDS library), for their effects on adhesion and cell morphology of fibroblasts. We identified nine compounds that reduced cell spreading and one compound (Clomipramine) that counteracted spreading inhibition elicited by Nogo-A`s functional Δ20-domain. Clomipramine was shown to also promote neurite outgrowth in primary cultured cerebellar neurons, suggesting a more general mechanism of action on cell spreading and neurite outgrowth in two different cell types. 

## Results and Discussion

### Screening Assay Design

To study the effects of a library of chemical compounds on cell adhesion we developed a low-cost screening approach using only freely available software and equipment available in most biological laboratories. 

**Figure 1:**
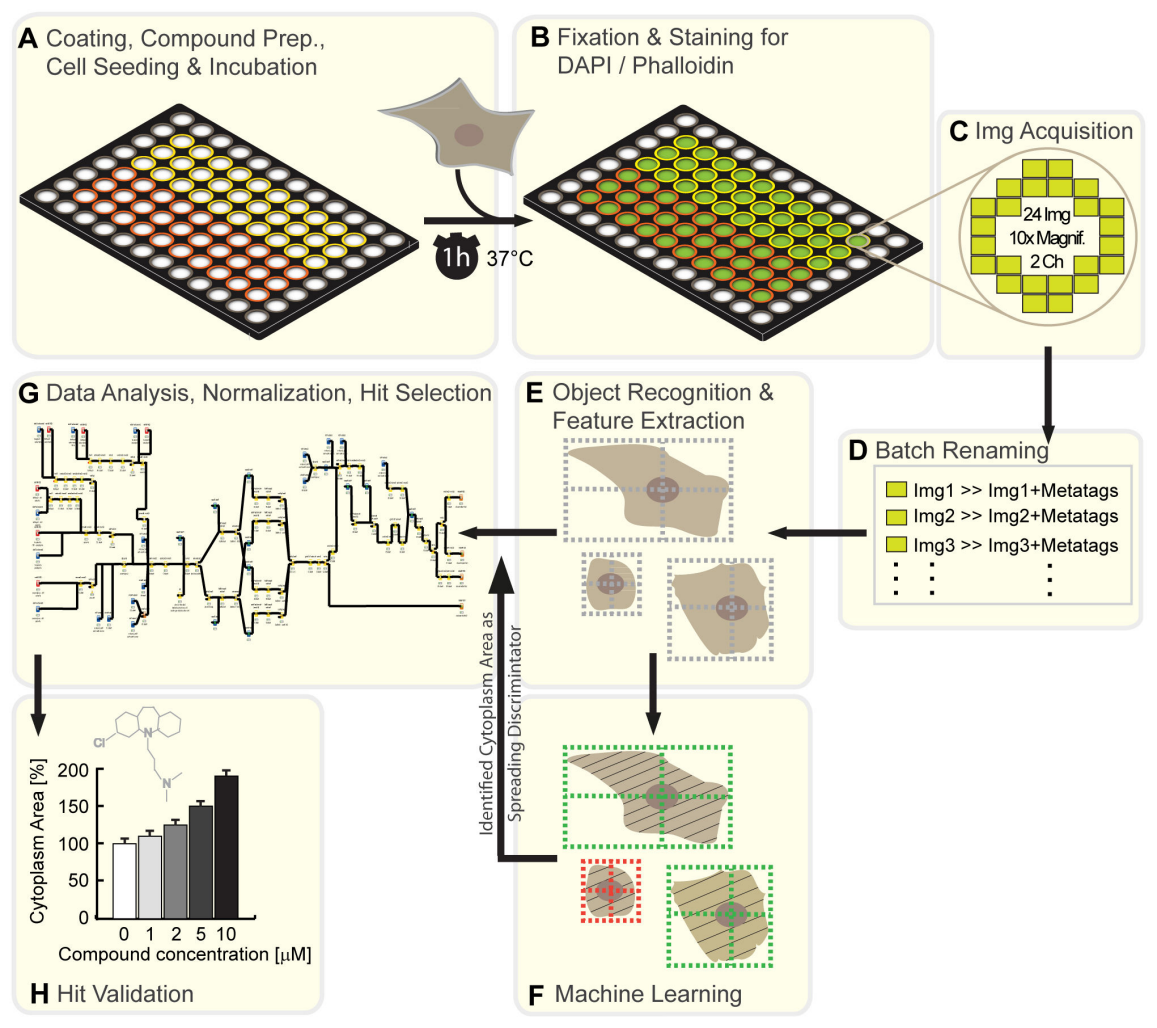
High Content Screen (HCS) for cell spreading of 3T3 fibroblasts. The major steps of the screen are illustrated: (**A**) Swiss 3T3 cells are incubated with different compounds in 96-well plates on two different substrates (yellow/red). Each column contains the same compound. The outer wells are left empty. (**B**) After 1h incubation at 37°C/5% CO_2_, cells are fixed with 4% paraformaldehyde in isotonic phosphate buffer and stained with DAPI (nuclei) and Phalloidin (F-actin). (**C**) 24 images are acquired per channel from each well using the 10x objective. No images are taken in the center or at the border of the well. (**D**) Image file names are being padded with meta-tags containing location and treatment information. (**E**) Using a custom developed software pipeline in ***Cell*** ***Profiler***, cell bodies and cell nuclei are automatically detected. Phenotypic features are extracted and stored in an SQLite database, annotated with its corresponding meta-tags and linked to its compressed image files. (**F**) The SQLite database can be imported into ***Cell*** ***Analyst***. Machine learning algorithms can be used to detect the most prominent cell features that are changed in a certain condition. Furthermore, it allows for an automated categorization of cells by their phenotypes (e.g. spread vs. unspread cells). (**G**) A data analysis pipeline was constructed in ***KNIME*** to automatically import, normalize and visualize the data. Scatter plots/matrices allow for outlier removals and hit selections. Pivot tables of normalized data / hit lists are generated and exported into .csv files for direct import into statistical analysis software. (**H**) All hits were validated in dose-response assays with 6 instead of 3 replicates.

**Figure 2:**
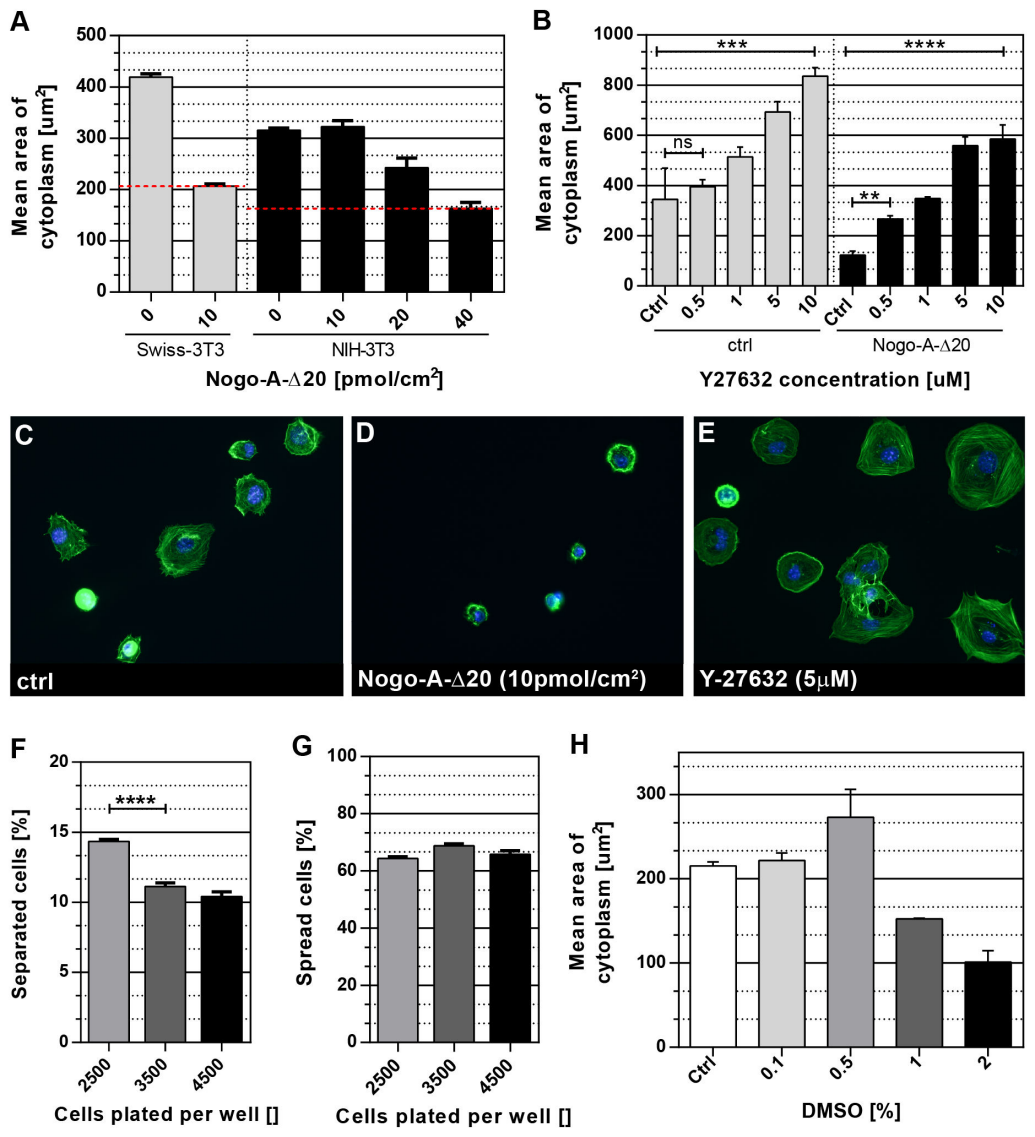
Assay optimization and validation. To ensure assay reliability different cell culture conditions were tested: (**A**) *Cell* *type*. Graph shows cell area after one hour of cell spreading on control vs. Nogo-A-Δ20 substrate for two different 3T3 fibroblast lines (red dotted lines mark IC_50_ of Nogo-A-Δ20). (**B**) *Positive control*. ROCK inhibitor (Y-27632) dose-response curve for 3T3 cell spreading on control (plastic) vs. Nogo-A-Δ20 substrate (10 pmol/cm^2^). (**C/D/E**) Representative pictures of cells incubated with either 0.1% DMSO (ctrl), 10 pmol/cm^2^ Nogo-A-Δ20 (inhibitory substrate) or 5μM Y-27632 (positive control). (**F/G**) *Cell Number*. The amount of imaged, well separated cells (cells without contact to neighboring cells) per total cells plated is shown in (F) while the number of cells characterized as spread per total cells plated is shown in (G). (**H**) *DMSO toxicity*. A dose-response curve for increasing DMSO concentrations on cell size (cytoplasm area) is plotted. *All experiments were performed at least in triplicate (n=3)*. *For all graphs: standard errors of the means are shown*. *Statistical* *analysis* *was* *performed* *in* ***GraphPad*** ***Prism6*** *using* *an* *ordinary* *One-Way* *ANOVA* *test* *followed* *by* *a* *Tukey multiple* *comparison* *test* or *by* *using* *an* *unpaired* *Student’s* t-test; p-values: ns>0.05; *<0.05, **<0.005, ***<0.0005, ****<0.00005 .

**Figure 3:**
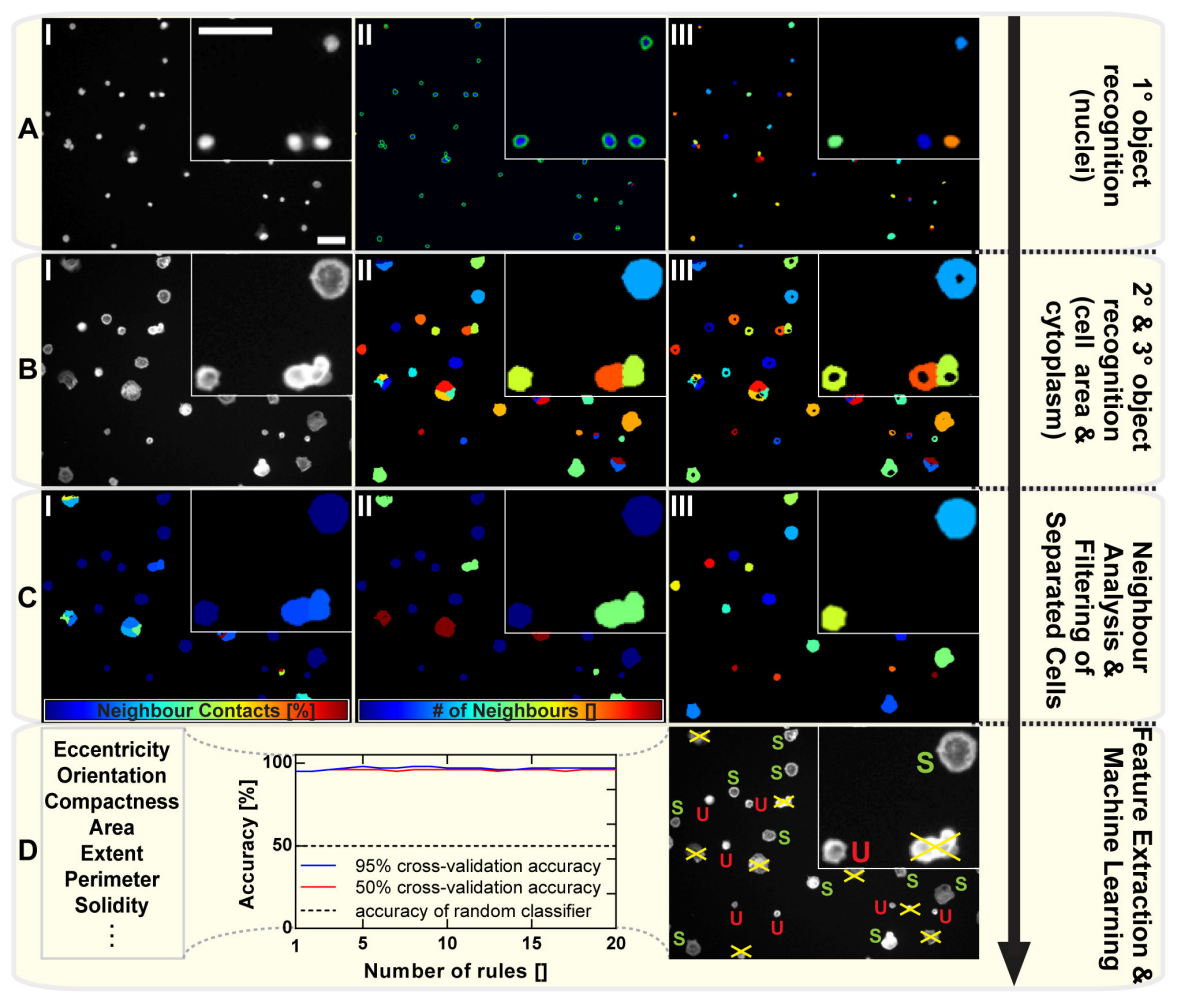
Data acquisition and feature extraction. A “High Content Screening” data acquisition pipeline was developed using the ***Cell*** ***Profiler*** software package: (**A**) *Primary* *object* *recognition*. DAPI stained cell nuclei (I) are detected and classified as primary objects (II). Nuclei touching the border, as well as nuclei outside the typical diameter range are excluded from the analysis (III). (**B**) *Secondary and tertiary object recognition*. Cell borders are detected in the Phalloidin channel (I) through a propagation function, that uses the DAPI nuclei (primary objects) as seeds to propagate outwards to the region of highest intensity (cell border). Again, cells touching the border of the image are excluded from analysis (II). Tertiary objects (cytoplasm) are generated by subtracting primary from secondary objects (III). (**C**) *Neighbor analysis and filtering of separated cells*. The amount of cell-cell interactions is measured by analyzing each cell’s fraction of membrane in contact with other cells (I) as well as the number of contacting neighbors (II). A filter is applied to extract all “separated cells” (no contact with other cells) (III). (**D**) *Feature extraction and machine learning*. All separated cells are subjected to a multi-parameter feature extraction. The information as well as compressed images of all channels is stored in an SQLite library and imported into ***Cell*** ***Analyst***. Here, machine learning algorithms are generated to automatically discriminate spread (S) from unspread (U) cells. Thereby, the algorithms are built on a subset of cells that are categorized manually. The plot displays the typical cross-validation accuracy of a 3-class classifier (using 20 rules) applied to such a subset. - Magnification: Calibration bars in A(I) are applicable to all images of A-C and correspond to 50µm.

**Figure 4:**
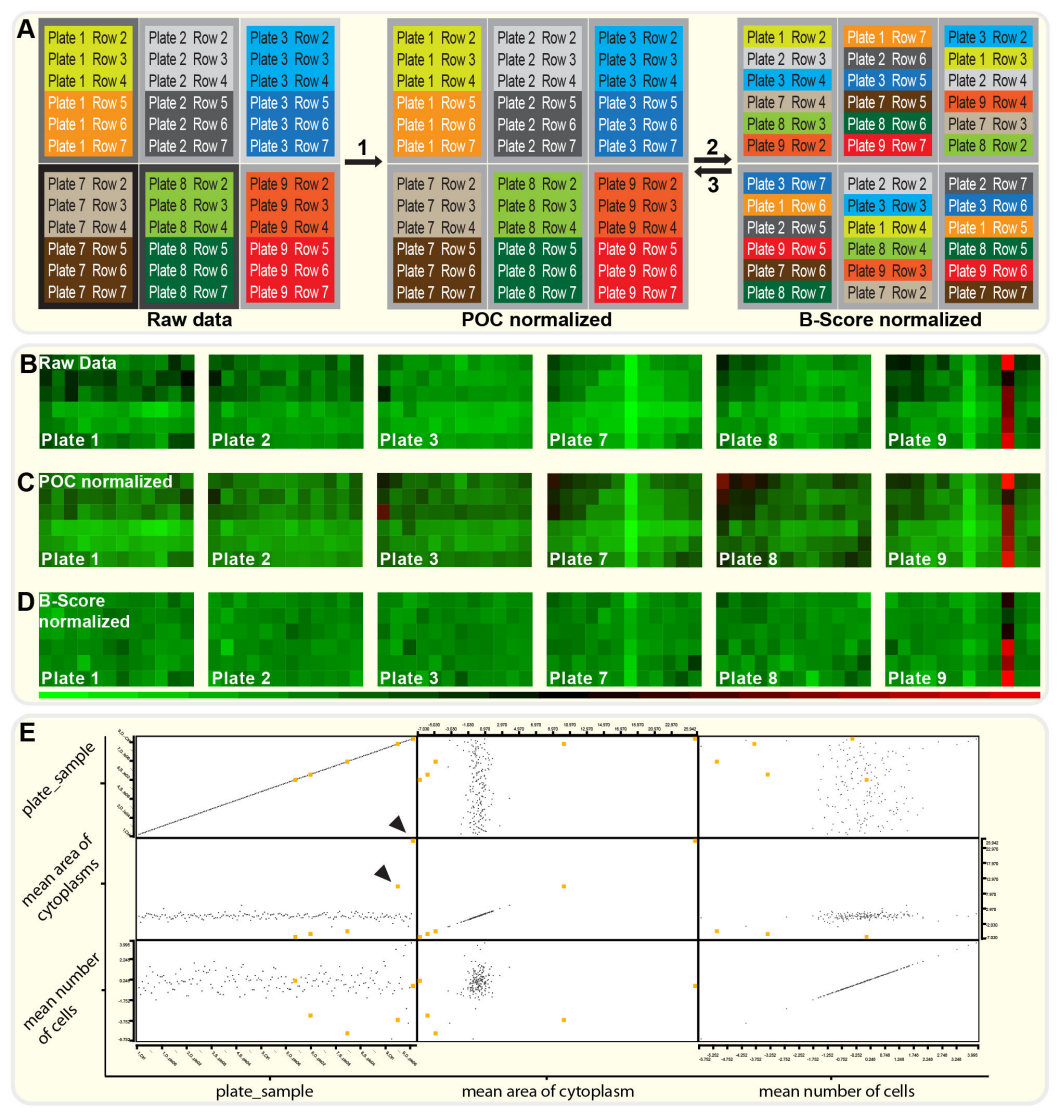
Data normalization and hit selection. A data analysis pipeline was developed using the ***KNIME*** software package. The steps of data normalization and hit selection are shown: (**A**) *Data* *normalization* *using* *virtual* *row* *shuffling*. A schematic representation of the data normalization procedure is shown for the three lower and upper plates of each experimental stack (9 plates total). First, a percentage of control (POC) normalization is used to normalize each plate to its corresponding DSMO control on ctrl substrate (1). Next, a virtual row shuffling algorithm is applied to allow for median averaging (B-Scoring): Hereby, rows are virtually being “shuffled” with corresponding rows from other plates of the experiment. It is made sure, that each row`s position on a virtual plate corresponds to its position on the original plate. Furthermore, only rows from plates in comparable stack positions are being mixed to prevent stack position effects to influence normalization (2). Finally, normalized data are reverse-shuffled to their original layout and subjected to hit selection (3). (**B**-**D**) Representative heatmaps (cytoplasm areas, increasing from green via black to red) are shown for raw data (B), POC normalized data (C) and B-Score normalized data (D). (**E**) *Hit Selection*. Hits are selected manually from scatter matrices generated in KNIME. Any compound, selected as hit in either plot is being marked yellow in all plots of the scatter matrix. All hits are automatically exported into hit lists for further analysis. Black arrowheads mark positive control (ROCK inhibitor Y-27632) on ctrl (left) and Nogo-A-Δ20 substrate (right).

Figure ***1*** depicts the major steps of the screen: First, 96-well plates were coated with the desired substrates/proteins overnight at 4°C. On the next day, compound stocks were diluted and transferred to the 96-well plates. 3T3 fibroblasts were added to the wells and incubated at 37°C (Fig.***1A***). After one hour cells were fixed with paraformaldehyde and stained with fluorescently labeled Phalloidin (F-actin marker) as well as DAPI (nuclear marker) (Fig.***1B***; Fig.***2C-E***). All plates were imaged using an imageXpress Micro HCS MD1 inverted epifluorescent microscope. 24 images were acquired per well and channel using the 10x objective. To ensure an even distribution of the cells we decided to follow an imaging layout as depicted in Figure ***1C***. Only images that were neither in the center nor close to the border of the well were acquired. Next, all images were renamed in an automated batch process (“ReNamer” freeware) to incorporate well-specific meta-tags such as coating condition, plate number or well position (Fig.***1D***). Using a custom developed processing pipeline within the ***CellProfilersoftwarepackage***, cell bodies and cell nuclei were detected, measured for features such as ‘cytoplasm area’ (total cell size minus nuclear area) or cell shape and stored in a database with its corresponding meta-tags (Figure ***1E***; Figure ***3***). While a first visualization of the raw data can be obtained in ***CellAnalyst***, this software package was mainly used to implement machine learning algorithms into the processing pipeline to discriminate spread from unspread cells (Figure ***1F***; Figure ***3***). The data from both CellProfiler as well as CellAnalyst were then fed into ***KNIME***. Within this software package we designed a complex data analysis pipeline that automatically imports, normalizes and visualizes the data (Figure ***1G***; Figure ***S3***). Furthermore it provides a visual output of the data and allows for hit selection of effective compounds. Cell spreading is analyzed. A detailed description of the data normalization and hit selection procedure can be found below (Figure ***4***). In a final step of this pipeline, all sorted and normalized data, as well as hit lists are exported to comma-separated value (*.csv) files that can be imported into a spreadsheet software of one’s choice or directly plotted in statistical software such as GraphPad PRISM. All hits were validated in follow up experiments with 6 instead of 3 replicates. Successful candidates of interest were finally tested in dose response assays to check for concentrations of highest functional activity as well as possible toxicity effects at higher doses (Figure ***1H***). 

### Assay Optimization and Validation

In order to ensure robustness of the assay we tested for different 3T3 cell lines, numbers of cells, numbers of replicates, fixation times as well as for DMSO toxicity and an optimized staining protocol. We furthermore tested the assay using both positive and negative controls for cell spreading (Figure ***2***, Table ***1***). While in general any cell type can be used with this protocol to study cell spreading, we were especially interested in 3T3 fibroblasts. Established in 1962 by Todaro and Green [14] the 3T3 cell line has rapidly become a standard fibroblast cell line in research. In the field of axonal regeneration it has played an important role in understanding the basic signaling mechanisms of the neurite outgrowth-inhibiting myelin protein Nogo-A. If incubated with the Nogo-A-Δ20 fragment, spreading and adhesion of 3T3 cells as well as neurons was reduced significantly (Figure ***2A,C,D***) [15]. Interestingly, in contrast to the Nogo-66 domain of Nogo-A, Nogo-A-Δ20 seems to signal independently of the Nogo receptor 1 (NgR1) since NgR1 is not expressed in 3T3 fibroblasts [15].

**Table 1:** Assay Optimization.

	**Optimized Condition**
**Cell type**	Swiss-3T3 fibroblasts
**Replicate number**	3
**Cell number**	2,500 per well
**Fixation time**	1h
**Nogo-A-Δ20 coating**	10pmol per cm^2^
**DMSO concentration**	no toxicity effects up to 0.5%
**Staining**	Phalloidin: 1:2000; DAPI: 1:10000

We first optimized the cell type for our spreading assay by comparing Swiss 3T3 cells to a second frequently used line, NIH-3T3 cells. While the latter cell type is easier to transfect, cell size after plating for 1 hour is about 25% smaller for NIH-3T3 as compared to Swiss 3T3 cells. Furthermore, responsiveness to anti-adhesive Nogo-A-Δ20 protein of NIH 3T3 in comparison to Swiss 3T3 cells was about 4-fold lower in NIH-3T3 than in Swiss 3T3 (Figure ***2A***). To increase the possible range of spreading-modulation and to reduce the amount of Nogo-A-Δ20 peptide needed, we decided to use Swiss 3T3 cells for our experiments. Figure ***2C*** shows a representative picture of Swiss 3T3 cells plated on plastic (ctrl) and Nogo-A-Δ20 substrate. Cells show a greatly reduced spreading phenotype characterized by reduced cytoplasm-areas and cell rounding.

The small, soluble GTPase RHOA is known to mediate the inhibitory effects of Nogo-A on neurite outgrowth and cell spreading [7,16]. The rho-associated protein kinase (ROCK) inhibitor *Y-27632* was therefore used as a positive control. It specifically enhanced cell spreading at low concentrations (500nM) on inhibitory Nogo-A-Δ20 substrate, but higher concentrations also significantly increased spreading on the control plastic substrate, arguing for a basal activation of the RHOA-ROCK pathway in spreading 3T3 cells and an increased activation after stimulation with Nogo-A-Δ20 as described before (Figure ***2B***; [16-18]). We decided to include the ROCK inhibitor at a concentration of 5μM in the screen to act as a strong positive control for cell spreading. A representative picture of this drugs’ effect at this concentration is shown in Figure ***2E***. Next, we tested for an optimal number of cells to plate per well. While 2500 cells per well yielded about 350 separated, individual cells (not touching any neighboring cells) per 24 images acquired for analysis, increasing numbers led to only a small increase in separated cells due to the increased exclusion of cells touching their neighbors (Figure ***2F/G***). To ensure enough room for enhanced spreading phenotypes and to reduce the number of cells needed for the screen, we decided to plate 2500 cells per well. To test for toxicity effects of Dimethyl Sulfoxide (DMSO) – the solvent of many compound stocks –spreading of Swiss 3T3 cells was tested in the presence of 0.1 up to 2% DMSO. Concentrations of 1% and above seemed to be toxic (decreased spreading), but 0.1% and 0.5% DMSO showed no inhibitory effect on cell spreading (Figure ***2H***). In the screen we used compounds at a concentration of 2μM in 0.2% DMSO, as well as compounds at a concentration of 5μM in 0.5% DMSO. The high DMSO concentration was used as a control for reduced spreading; the screen was thus successfully validated with controls for enhanced as well as decreased spreading. Finally, the “strictly standardized mean difference” (SSMD), denoted as β, was used for statistical evaluation of the screen’s reliability [19]. With the ROCK inhibitor acting as the positive control and 0.2% DMSO as the negative control (no change in cell spreading), SSMD values of β>7 were consistently achieved for changes in “cytoplasm area” (three separate experiments; plate layout: Figure ***1***; assay conditions: Table ***1***). Following the interpretation guidelines proposed by [19] an SSMD value above 7 – even in assays with strong positive controls – demonstrates an “excellent” assay quality.

### Data Acquisition and Feature Extraction

After the spreading assay had successfully been developed in a 96 well-plate format, a major challenge remained: the development of a powerful but flexible data acquisition and analysis pipeline. To enable use and adaptation for differing biological questions and laboratories, we decided to build a pipeline based on the following criteria: 1) software must be free of costs, 2) software must have a graphical user interface, 3) pipeline must be editable to be adaptable to each laboratory according to its needs, 4) software has to support the reduction in pipetting steps through an intelligent data normalization algorithm. 

After careful evaluation we decided to construct the pipeline within two software packages that allowed us to meet all of the criteria listed. The ***CellProfiler/Analyst*** software package was used to construct the data acquisition pipeline, while ***KNIME*** was used to build the data integration, normalization and analysis pipeline. 

Figure ***3*** highlights the most important steps during data acquisition. To ensure a consistently working analysis and to prevent object recognition artifacts we first applied an illumination correction and a “rescale intensity function”. The corrected images were then used to detect all nuclei (DAPI channel) and classify them as primary objects (Figure ***3A***). Nuclei touching the border, as well as ‘nuclei’ outside of the typical diameter (mostly staining artifacts) range were excluded. Next, we applied an Otsu thresholding algorithm to the phalloidin-channel images taking into account the minimum and maximum values in the image and log-transforming the image before calculating the threshold. In this way, thresholding is reliably performed independent of the image area covered by cells. To prevent a misdetection of clumped cells as single cells, we furthermore installed a propagation function, that uses the DAPI nuclei (primary objects) to detect cell borders by propagating outwards to the region of highest intensity (Figure ***3B***). Again, cells touching the border of the image were excluded from the analysis. By subtracting the primary (areas of nuclei) from the secondary objects (total cell areas), we generated the tertiary objects (cytoplasm areas) to be used in the subsequent analysis (Figure ***3B***). In this way we were able to focus on the cytoplasmic area without diluting the measurements with the nuclear area that is unchanged in both, the spread and the unspread condition. As a result, differences between rounded and flattened cells were increased enabling us to resolve smaller changes. In a next step we made sure that only single cells were being used for analysis, since cell aggregation leads to unpredictable effects on the cells’ phenotypes. For that, a module was implemented to measure the number of each cells’ neighboring cells (Figure ***3C***). Also, the percentages of the cells’ membranes in contact with neighboring cells were measured. We decided to set a cutoff of 0 for both measurements to ensure analysis of only single cells. Importantly however, the cutoff may easily be adapted to ones’ choice. The single or “separated cells” – as we termed them – were filtered out (Figure ***3C***) using this cutoff and subjected to an extensive feature extraction for attributes such as area, compactness or perimeter length (Figure ***3D***). Finally, several export modules were implemented to export all measurements to comma separated value (csv) files as well as a SQLite database. Furthermore, images of all channels as well as all primary and secondary objects were exported as compressed Jpeg image files and linked to the database. 

### Machine Learning: Guided Phenotype Discrimination

We aimed to develop a method that can automatically perform cellular size or shape change analyses automatically using machine learning algorithms for phenotype discrimination. Such a process also enables to answer the question of what features are mainly characterizing a given phenotype observed. 

We used the ***CellProfilerAnalyst*** software package that is able to directly access the SQLite database as exported from the CellProfiler Pipeline.

Using the classifier module, 100 cells were randomly picked from the experiment and categorized using personal judgment to be either spread or unspread (Figure ***3D***). With a maximum number of 20 rules, the classifier was then trained to discriminate these two categories based on their features as extracted in CellProfiler. As expected, the most promising discriminator for cell spreading was found to be the cytoplasm area which was used as a direct read out for cell spreading in the further analysis. Instead of a “digital” yes or no to cell spreading, the “analog” analysis of cell area allowed the detection of much smaller changes in cell spreading. Nevertheless, the data obtained in CellProfiler Analyst was fully comparable to the one from manual evaluation using light microscopy.

Furthermore, a phenotypic discrimination using machine learning algorithms provides another advantage: Instead of solely analyzing compounds for their effects on cell spreading (area), it is possible to quickly apply an unbiased morphological analysis able to detect changes in cell characteristics such as roundness, compactness, solidity, eccentricity and all other features extracted in the CellProfiler pipeline (Figure ***3D***).

### Data Normalization

One of the most important determinants for a successful pharmacological screen is its’ statistical robustness. While robustness can be enhanced through an increased number of replicates or a randomized plate layout, a successful screen needs to balance robustness with work-load and costs. Since our aim was to develop a screen for lab environments without access to automated liquid handling stations, a reduction in manual labor was considered a high priority. We developed a plate setup that enabled us to use multi-channel pipettes for almost all steps throughout the protocol, thus reducing manual labor and error rates through prevention of single-well pipetting steps. 

As demonstrated in **s*upporting*** Figure ***S1C*** and described in more detail in the method section, the outer rows of all assay plates were left empty. No cells were plated, but they were filled with medium and DMSO to ensure a homogeneous environment for all wells used in the experiment. While cells in the upper three rows were used for controls (ctrl; 3 replicates), the lower three rows were coated with the experimental substrate (Nogo-A-Δ20 protein; 3 replicates).

A multi-channel pipette allowed us to transfer 9 different compounds + 1 DMSO control directly from the stock plate via a pre-dilution plate into the final assay plate. (Figure ***S1***)

While this plate setup certainly reduces manual labor it comes with a trade-off: missing randomization increases the impact of intra- and inter-plate effects requiring an intensive normalization. 

We decided to implement two normalization steps into the analysis pipeline by using the ***KNIMEsoftwarepackage***. First, a percentage of control (POC) normalization removed inter-plate differences by normalizing the data of each plate to the plate’s corresponding DMSO control on ctrl substrate. Next, we decided to use median averaging (B-Score) algorithms on each plate to reduce intra-plate effects (Figure ***4B***).

The B-Score, analogous to the Z-Score, assumes that most compounds are inactive and can serve as controls. Compared to the Z-Score however, it uses an index of dispersion that is more resistant to the presence of outliers and more robust to differences in measurement error distributions of the compounds [20].

While the POC normalization was quickly implemented, B-Scoring implementation proved to be challenging since the plate layout contradicts the B-Scorings’ basic assumption of having only few single wells with “hits” (compounds modulating spreading). Instead, each hit was present at least three times on each plate. On top of that, spreading was reduced on half of the plate due to the Nogo-A-Δ20 coating.

To nevertheless allow B-Scoring normalization, we developed the strategy of “virtual row shuffling” using a pipeline built within KNIME (Figure ***S3***). Figure ***4A*** illustrates the basic concept of this method: After POC normalization, rows are being virtually “shuffled” with corresponding rows from other plates of the experiment. It is made sure, that each row`s position on a virtual plate corresponds to its position on the original plate. Furthermore, only rows from plates in comparable stack positions are being mixed to prevent stack position effects to influence normalization (each experimental stack consisted of 9 plates in the order of plate 1-9 from bottom to top).

The resulting virtual plates contain each compound only once. Also, the same coating is present throughout the whole plate (Figure ***4A***). The B-Scoring algorithm can thus be applied to correct for row and column effects. After successful implementation of this technique in the analysis pipeline, the normalized data was shuffled back (“reverse-shuffling”) to its original plate layout and visualized using the heat maps module in KNIME (Figure ***4B-D***). As visible in Figure ***4D***, normalization effectively reduces plate position effects on cytoplasm area. Also apparent is the normalization of coated and uncoated conditions to a common baseline. This allows not only for an easier hit selection but also provides a fast insight into how effective a certain compound is in modulating spreading in the coated vs. uncoated conditions. 

### Hit Selection and Data Export

While the whole data analysis pipeline was developed to run in a fully automated manner, we also decided also to implement a tool that would allow for a fast and very efficient semi-manual hit selection. We implemented interactive scatter plots as well as scatter matrices into the pipeline. Figure ***4E*** shows an example of a scatter matrix that automatically is constructed from plate and sample identifiers, the cytoplasm areas, as well as the number of cells for one of our experiments. Here, the plot of cytoplasm area vs. plate/sample identifier, clearly demonstrates the stability of the baseline. Hits can be selected individually or in groups and are marked in orange. Black arrowheads show the effect of the positive control (Rock inhibitor Y-27632) in control (left) and Nogo-A-Δ20 (right) conditions. Alternatively, hits can also be selected from the “cytoplasm area vs. cytoplasm area” or “cell numbers vs. cell numbers” plots. 

All normalized measurements as well as hit lists are finally exported into comma separated value files to be read into a spreadsheet software of one`s choice. We furthermore implemented an automated pivot table module to prepare data for statistical software such as GraphPad Prism. Without any changes the data can be copied into such software to plot data graphs and perform statistical analyses with the push of a button. 

### Adaptation of pipeline to other experimental setups or analysis methods

While the described pipeline was built to analyze the effects of compounds on fibroblast spreading and to plot the mean areas of the cytoplasms, it is easily adaptable to other experimental setups or analysis methods. An example is given for siRNA based screens in ***supporting*** Figure ***S2***: Here, instead of using compounds, 3T3 cells are treated with the transfection/nucleofection marker siGlo, a non-RISC engaging molecule that localizes to the nucleus to provide visual indication of successful siRNA transfection/nucleofection. Only cells positive for siGlo are to be analyzed. To achieve this, the pipeline is modified as depicted (Figure ***S2***). After primary (DAPI) and secondary (Phalloidin) object recognition (Figure ***S2A/B***), the siGlo channel is read in using a second primary object recognition module. A manual threshold is applied to exclude background signals and staining artifacts (Figure ***S2C/D***). Finally, cells are not only analyzed for contacts to neighbors but also for co-localization with siGlo. Only separated, co-localized cells are finally filtered out to be used for analysis (Figure ***2E***). The ***modifiedCellProfilerpipeline*** can be downloaded as ***supporting*** File ***S4***.

Next to the possibility of adapting the pipeline to general changes in the experimental design, data analysis is also highly customizable. An example is shown in ***supporting*** Figure ***S2F-G***. Instead of plotting the mean areas of the cytoplasms, an object based analysis can be used to plot cell areas in bins of defined sizes (Figure ***S2G***) or, by using statistical software such as GraphPad Prism, as cumulative frequency distributions (Figure ***S2H***). The object based analysis of two independent experiments on control as well as Nogo-A-Δ20 substrates showed almost identical distribution profiles of cell areas, indicating a reliably working pipeline. (Figure ***S2F-G***)

### NINDS-II Compound Screen Reveals Modulators of Cell Spreading in Swiss-3T3 Cells Plated on Nogo-A-Δ20 vs. Control Substrates

**Figure 5:**
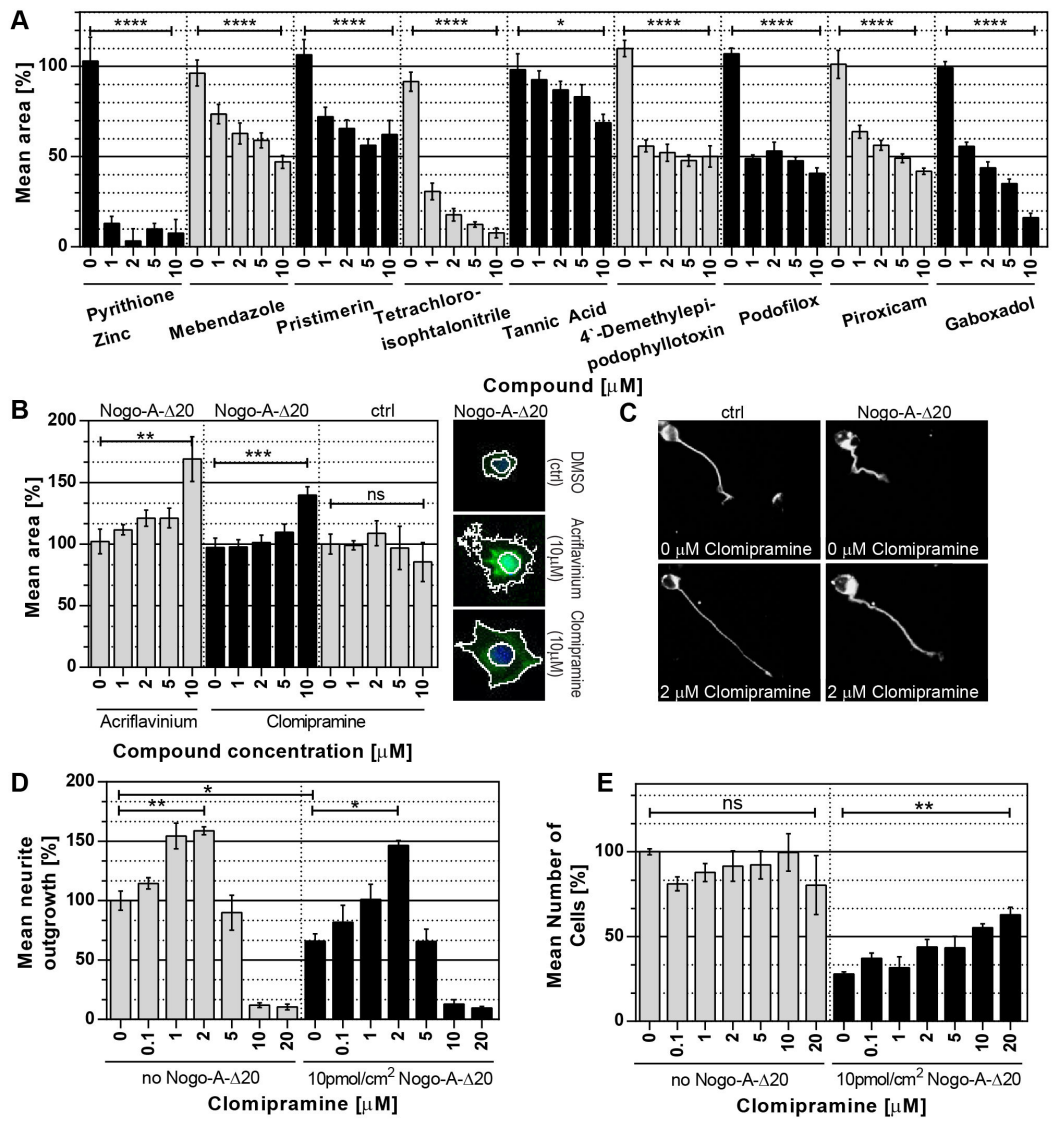
Hit validation and neurite outgrowth assay. Nine of the strongest negative hits (inhibitors of cell spreading) as well as 2 of the strongest positive hits (enhancers of cell spreading) validated in dose-response assays are shown (6 replicates per condition): (**A**) *Validated* *negative* *hits*. Of ten compounds tested, nine were validated to significantly decrease cell spreading. For these, the cytoplasm area is plotted against the compound concentration. (**B**) *Validated positive hits*. Of five compounds tested, two were initially validated to significantly enhance cell spreading. Further evaluation excluded Acriflavinium but not Clomipramine for its autofluorescence since it showed to interfere with the secondary object recognition (representative pictures). (**C**-**E**) *Neurite Outgrowth Assay*. Clomipramine was further tested for its effects on neurite outgrowth in a pure culture of cerebellar granule neurons (CGNs; postnatal day 7). Neurite outgrowth (D), as well as cell adhesion (E) was analyzed in dose-response assays on control as well as Nogo-A-Δ20 substrates. Representative images of CGNs with and without 2μM Clomipramine on these two substrates are shown in (C). *All experiments were performed at least in triplicate*. *For* *all* *graphs: standard* *errors* *of* *the* *means* *are* *shown, N= 6* (A,B) *or 3* (D,E)*, respectively; Statistical* *analysis* *was* *performed* *in* ***GraphPad*** ***Prism6*** *using* *an* *ordinary* One-Way ANOVA *test* *followed* *by* *a* *Tukey multiple* *comparison* test; p-values: ns>0.05; *<0.05, **<0.005, ***<0.0005, ****<0.00005 .

The established protocol was applied to a medium-throughput screen using the “National Institute of Neurological Disorders and Stroke” (NINDS-II) compound library that contains 1040 compounds of which three quarters are FDA approved [21]. Using the screen we initially identified 10 “negative hits”, i.e. compounds that reduce spreading (Table ***2***). There was no selectivity with regard to the control or Nogo-A substrate. We also found 5 “positive hits”, i.e. compounds increasing spreading on control and/or Nogo-A substrate (Table ***3***). All compounds scored as hits as well as 2 randomly picked compounds that did not show an effect in the screen (Table ***4***) were further validated using dose response assays. 9 out of 10 compounds initially scored to possess spreading reducing effects on both, Nogo-A-Δ20 as well as control substrate were validated. 5 of these 9 compounds, showed a dose dependency in the validation experiments (Figure ***5A***). Literature research revealed several modes of actions in five of the nine compounds that are known to influence cell spreading. Three compounds have been described to disrupt microtubule polymerization (Mebendazole, 4`-Demethylepipodophyllotoxin and Podofilox [22,23]) – a process known to be detrimental for cell spreading [24]. Two other compounds either inhibit prostaglandin synthesis via COX-2 (Piroxicam [25]) or via disruption of β1-integrin-ligand binding (Pristimerin [26]). Both processes are known to be implicated in interfering with cell spreading [26,27]. While this further demonstrates that the screen is working reliably, it is important to bear in mind that reduction in cell area can also be the result of toxicity. An example for this could be Pyrithione Zinc, a DNA synthesis inhibitor that scored as a negative hit but is known to be cytotoxic in 3T3 cells at the concentrations tested [28]. In our experience a good first indication for a toxic effect of a compound is given by its dose response profile. While spreading reducing compounds often reach a plateau phase with increasing concentrations (as seen in Figure ***5A*** for Pristimerin, 4`-Demethylepipodophyllotoxin and Podofilox) before becoming toxic eventually, purely toxic compounds (as seen in Figure ***5A*** for Pyrithione Zinc) do not show this biphasic effect. For the three validated negative hits Tetrachloroisophtalonitrile, Tannic Acid and Gaboxadol further tests will be needed to discriminate between toxicity and cell spreading inhibition. 

**Table 2:** NINDS Screen - Negative Hits.

**Compound**	**Molecular Formular**	**Therapy**	**Source**	**Normalized Area**	**Validated?**
PYRITHIONE ZINC	C10H10N2O2S2Zn	antibacterial, antifungal, anti-seborrheic	synthetic	-14.4	YES
GABOXADOL HYDROCHLORIDE	C6H9ClN2O2	GABAa agonist, GABAc antagonist	synthetic; THIP hydrochloride	-9.6	YES
TANNIC ACID	C76H52O46	nonspecific enzyme/receptor blocker, inhibits corrosion, food applications	principal constituent of tree galls,	-9.18	YES
BENZTROPINE	C21H27NO5S	anticholinergic	synthetic	-8.39	NO
PRISTIMERIN	C30H40O4	decreases β1-integrin-ligand affinity, antineoplastic, anti-inflammatory, antiviral	*Celastrus* and *Maytenus* spp	-7.2	YES
PIROXICAM	C15H13N3O4S	COX inhibitor, anti-inflammatory	synthetic	-6.53	YES
4'-DEMETHYL-EPIPODOPHYLLOTOXIN	C21H20O8	inhibits microtubule assembly, antineoplastic	semisynthetic	-6.09	YES
PODOFILOX	C22H22O8	inhibits microtubule assembly, antineoplastic	Podophylotoxin Podophylum peltatum	-5.13	YES
TETRACHLOROISO-PHTHALONITRILE	C8Cl4N2	antifungal	synthetic	-5.03	YES
MEBENDAZOLE	C16H13N3O3	inhibits microtubule assembly, anthelmintic	synthetic	-4.95	YES

**Table 3:** NINDS Screen - Positive Hits.

**Compound**	**Molecular Formular**	**Therapy**	**Source**	**Normalized Area**	**Validated?**
ACRIFLAVINIUM HYDROCHLORIDE	C14H14ClN3	anti-infective (Human African trypanosomiasis, fungal infections), intercalating agent, anti-cancer activity	synthetic	9.22	YES *
CACODYLIC ACID	C2H7AsO2	anti-eczema, dermatologic, herbicide	synthetic	5.81	NO
SIBUTRAMINE HYDROCHLORIDE	C17H27Cl2N	anorexiant, antidepressant, uptake inhibitor (5HT, norepinephrine, dopamine)	synthetic	4.39	NO
COTININE	C10H12N2O	antidepressant, nootropic and anti-psychotic-like effects	*Nicotiana tabacum*	3.62	NO
CLOMIPRAMINE HYDROCHLORIDE	C19H24Cl2N2	antidepressant	synthetic	2.73	YES **
FOSCARNET SODIUM	CNa3O5P	antiviral	synthetic	2.63	NO

**Table 4:** NINDS Screen – inactive control compounds.

**Compound**	**Molecular Formular**	**Therapy**	**Source**	**Normalized Area**	**Validated?**
TRYPTOPHAN	C11H12N2O2	antidepressant, nutrient; LD50(rat) 1634 mg/kg ip	many plants, animal protein	0.19	YES
PROMETHAZINE HYDROCHLORIDE	C17H21ClN2S	antihistaminic, sedative	synthetic	0.59	YES

By testing the 5 “positive hits” in dose response assays we were able to validate 2 compounds (Figure ***5B***). Acriflavinium, however, exhibited a strong autofluorescence (Figure ***5B***) that led to the misdetection of a seemingly bigger cell area. This caveat can be controlled for by always cross comparing the screening data with the raw images (which can easily be done using Cell Analyst as described earlier). Interestingly Clomipramine, a tricyclic antidepressant, was the only compound identified in the screen able to reduce Nogo-A-Δ20`s effect on adhesion of Swiss-3T3 cells without affecting cell adhesion on the control substrate. Via dose response assays this specificity was further validated (Figure ***5B***). By using concentrations of up to 10μM we were in line with previous *in vitro* studies [29-38]. Additionally, 10μM is the therapeutic concentration often present in brain and plasma for tricyclic antidepressants [39]. 

### Clomipramine diminishes Nogo-A-Δ20-induced cell spreading and neurite outgrowth inhibition in highly purified Cerebellar Granule Neurons

We isolated highly purified cerebellar granule neurons from P7 Wistar rats and plated them on Nogo-A-Δ20/poly-L-lysine (PLL) or on a PLL control substrate for 24h. In the absence of clomipramine, outgrowth on Nogo-A was only 60% of that on PLL (Figure **5C/D**). Clomipramine at 0.1 - 2 uM strongly stimulated outgrowth, interestingly on both substrates. Values at 2 uM were almost similar on PLL or on the Nogo-A substrate, suggesting that, in addition to a strong growth stimulatory effect, Clomipramine also counteracted the growth inhibition of Nogo-A-Δ20. Similarly, the drug promoted neuronal adhesion to the Nogo-A substrate (at higher concentrations), without affecting adhesion to PLL (Figure ***5E***). D). Clomipramine is known to inhibit the reuptake of serotonin and norepinephrine but also to target a variety of other molecules [40]. A direct correlation between functional effects and underlying signaling events is, therefore, difficult.

## Conclusions

We developed a freely available, open source high content screening method to investigate cell spreading on different substrates in the presence of hundreds to thousands of compounds. This method was validated and used to detect small molecules that are able to influence cell adhesion and cell spreading of Swiss-3T3 cells in general and/or selectively modulate cell spreading and adhesion on Nogo-A-Δ20. 

Several known compounds, including drugs which interfered with microtubule assembly, were found in the NINDS-II library to inhibit fibroblast spreading, while a tricyclic antidepressant, Clomipramine hydrochloride, enhanced spreading of 3T3 cells on the inhibitory substrate Nogo-A-Δ20 and cerebellar neuron fiber growth on PLL and Nogo-A-Δ20. 

Importantly, except for an automated sampling fluorescent microscope, only routine lab equipment was employed in this assay. However, this screening method can be scaled up by using automated liquid handling systems to high throughput dimensions. 

Common alternative methods for investigating cell adhesion and spreading mostly require the researcher to manually acquire and analyze images of cells. This procedure is not only very slow and thus unsuitable for investigating high numbers of compounds, but also prone to human error. Other widely used methods for scoring adhesion are solely based on counting adherent cells before and after treatment. This is usually achieved by either labeling cells with a dye before counting them using a light microscope/plate reader or by measuring the absorbance of solubilized cells using a spectrophotometer [41]. While this approach is much faster compared to manually scoring of spread and unspread cells, it still lacks sensitivity. Subtle effects on adhesion, often resulting in changes of cell area, cannot be resolved. Consequently, the information obtained via the classic methods is mostly of qualitative instead of quantitative nature. Instead of subjectively classifying by eye whether a cell is spread or unspread or whether it is attached or not attached, the herein presented screening method allows for a detailed and standardized analysis of cell area and a wide range of additional phenotypic characteristics. 

We abdicated commercially available screening software solutions such as Metamorph (Molecular Devices) or Cellomics (Thermo Scientific), since these – due to their high purchasing costs – are mostly only available in industry laboratories or in specialized academic screening centers, offering fee-based services that can be very expensive and thus not ideal for many cell biology labs on a regular basis. Furthermore, commercially available analysis software packages are often limited in their adaptability, mostly unable to implement machine learning algorithms or requiring the use of an expensive screening microscope instead of being compatible to much cheaper and more widely distributed microscopes set-ups with programmable robotic stages. 

A variety of possible applications of the presented screening method are easily conceivable. Adjustable parameters include a wide range of possible cell types, the use of different adhesion modulating substrates or the adaptation of the multi-parameter readout criteria. Furthermore, we demonstrated the possibility to use this method for screening siRNA instead of compound libraries. 

In summary, we successfully developed and validated a highly customizable, versatile and cost effective screening pipeline to study cell adhesion and cell spreading without the need of expensive and specialized screening equipment. These methods will enable laboratories not routinely employing screens in their daily work to investigate the effects of a wide range of different compounds or siRNAs on adhesion, spreading and cytoskeleton-modulating molecules.

## Materials and Methods

### Ethics Statement

All experiments involving animals were performed with the approval of and in strict accordance with the guidelines of the Zurich Cantonal Veterinary Office. All efforts were made to minimize animal suffering and to reduce the number of animals required.

### Assay Development

#### Cultivation of Swiss-3T3 Cells

Swiss-3T3 (ATCC) and NIH-3T3 cells (ATCC) were maintained in DMEM (61965-026, Invitrogen) supplemented with 10% newborn calf serum (Invitrogen) and cultured in a 95% humidified incubator at 37°C with 5% CO_2_. 

New Swiss-3T3 cells were taken into culture regularly (every 5-10 passages) to prevent phenotypic changes in between cells used for experiments. 

#### Protein purification

His-/T7-tagged Nogo-A-∆20 protein containing amino acids 544-725 of rat Nogo-A was purified as described previously [15]. Briefly, BL21/DE3 *E. coli* were transformed with the pET28 expression vector (Novagen) containing the sequence of the recombinant protein and cultured at 37°C until an OD of 0.8 AU. 1 M IPTG was added for 2 h at 30°C to induce protein expression. After cell lysis with BugBuster Protein Extraction Reagent (Novagen) the fusion protein was purified using Co^2+^-Talon Metal Affinity Resin (Takara Bio Inc.).

#### Compound Library and Preparation of Stock Plates

The NINDS-II compound library was provided through a grant from The British Medical Research Council and consists of 1040 compounds of which three quarters are FDA approved [21]. It was originally compiled by MicroSource Discovery Systems for the National Institute of Neurological Disorders and Stroke (NINDS), the Huntington’s Disease Society of America (HDSA), the Amyotrophic Lateral Sclerosis (ALS) Association, and the Hereditary Disease Foundation (HDF) [21]. The library was shipped in thirteen 96-well plates, each containing 80 compounds with a total volume of 20ul and a stock concentration of 1mM in DMSO. The plates were stored at -80°C and were protected from light at all times possible. To reduce degradation the compounds were aliquoted into three 384-well plates (“mother plates”) with 6ul per well. The plate layout and pipetting scheme is depicted in ***supporting*** Figure ***S1***. Briefly, each row contained 9 wells of distinct compounds and 3 wells of DMSO ctrls. This way, one row was used to provide compounds for one final assay plate (“baby plate”). All nine rows of each 384-well mother plate thus provided compounds for nine final assay plates that were screened in one experiment.

#### Spreading Assay

The NINDS compounds were screened on Swiss-3T3 fibroblasts for their influence on cell-spreading. The assay was divided into 13 sub-experiments each testing 80 compounds, giving rise to 1040 tested compounds. Each sub-experiment consisted of nine 96-well assay plates, each of which testing 9 different compounds (Figure ***S1***). 

Each sub-experiment was performed as follows: The day before the assay, five 90% confluent 10cm dishes of Swiss-3T3 cells were split 1:2 using 0.05% Trypsin giving rise to 10 dishes of about 90% confluency the next day. In addition, an overnight Nogo-A-∆20 coating was performed on the nine 96-well assay plates. Three rows were incubated/coated with 50ul of 100nM Nogo-A-∆20 (rows E-G), and three rows (rows B-D) were incubated with 1x PBS to serve as controls (Fig.***S1C***). The coating was done on ice using pre-cooled 1x PBS to dilute the Nogo-A-∆20 stock solution. To reduce human error electronic multichannel pipettes (Rainin, inc. USA) were used for liquid handling throughout the experiments. The plates were immediately incubated overnight at 4°C and washed three times with 1x PBS shortly before the experiment.

Pre-dilutions (“daughter plates“) of the compounds were prepared shortly before usage in the experiment yielding 5x stocks for the final assay plates. 20ul of each pre-dilution was then transferred into the final assay plates (“baby plates“). The final compound concentration was increased from 2 to 5uM in sub-experiment 8-13 to test for a possible increase in hits by varying compound concentrations. 

Cells were prepared as follows: Ten 90% confluent 10cm plates were washed with 5ml of pre-warmed 37°C 1x PBS and 5ml of 1x PBS-EDTA was added for 15 minutes at 37°C to detach the cells. The cells were resuspended, pelleted at 700g for 6 minutes and counted using a hematocytometer. 80ul containing 2000 cells were plated per well and mixed with 20ul of the 5x compound stocks.

From sub-experiment 7 on, the Rock inhibitor Y27632 (Sigma, Y0503-1MG) was added as a positive control in column 10. 100ul of media were added into all empty wells of the plate to reduce intra-plate effects and ensure an evenly humidified environment. The plates were immediately placed in the 37°C incubator in an ordered stack with plate 1 on top. 

#### Fixation

After 1h, the cells were fixed for 20 minutes with 100ul of prewarmed 8% PFA in phosphate buffered saline (PBS). Cells were fixed in the order of plating to ensure equal incubation times and washed three times using 50ul PBS. 

#### Immunocytochemistry

Cells were blocked for 1 hour at room temperature using 100ul per well of PBS containing 0.3% triton X-100, 0.004% fish skin gelatin (Invitrogen) and 2% normal goat serum. The blocking buffer was exchanged against 50ul of fresh buffer containing DAPI (1:10,000, Invitrogen) and A488-coupled Phalloidin (1:2000, Invitrogen). Plates were incubated overnight at 4°C, washed two times using 1xPBS and stored at 4°C under protection from light until further use. 

#### Fluorescence imaging

The plates were imaged using an ImageXpress Micro HCS MD1 inverted epifluorescent microscope (Molecular Devices). 24 images were acquired per well. No images were taken in the center or close to the border of the well to ensure an even distribution of cells. Approximately 1h20min was needed to image one plate. The z-value and exposure time was readjusted between each plate to adjust for plate-to-plate variation with respect to different plate heights and staining efficiencies.

#### Neurite Outgrowth Assay

Cerebellar granule neurons from P7 Wistar rats were purified as described in [42]. Briefly, freshly isolated P7 rat cerebelli were dissociated using 1% trypsin. The resulting cell suspension was layered on top of a 35% / 60% percoll gradient and was centrifuged for 12min at 2000g. The interphase between the two percoll layers, containing the cerebellar granule neurons was carefully removed and preplated for 1h in a 100mm dish (TPP). The non-adherent fraction containing mostly cerebella granule neurons was collected and 100,000 cells were plated per well of a 4-well dish (Greiner) coated with 30ng/cm^2^ Poly-L-Lysine (PLL) with or without varying concentrations of Nogo-A-Delta-20.

After 24h of incubation at 37°C and 5% CO_2_, the cells were fixed using 4% PFA and stained with anti-β3III-Tubulin and DAPI. Images of the neurons were acquired at 10x magnification in an automated fashion using a modified Axioskop 2 microscope (Zeiss). The numbers of attached cells as well as the mean neurite lengths (total outgrowth divided by number of cells) were quantified using MetaMorph (Molecular Devices). Each experiment was performed in triplicate and the data were normalized to control and plotted as average ± SEM.

### Bioinformatics

All Analysis steps were performed using freely available software packages and custom designed streamlined pipelines. All setting files and pipelines can be downloaded from the supporting information section to be modified for personal use. Software packages are available from sources as stated below.

#### File preparation

To prepare files for the analysis pipeline, all acquired images were renamed using the freely available ***ReNamerUtility*** (http://www.heise.de/download/renamer-1151787.html) in batch mode. In this step also all meta data tags to be used in later analysis were incorporated in the file name. The settings file for the batch renaming process is provided as ***supporting*** File ***S1***. 

#### Image normalization

To guarantee consistent and reliable object recognitions throughout the experiments and within each field of view, images were analyzed using the following custom build pipeline (***supportingFileS2***) in the freely available ***CellProfiler2.0*** software (www.cellprofiler.org):

First, all phalloidin images were averaged to detect uneven illuminations throughout the field of view. This illumination function was then applied to every image to correct for shading. Next, a “rescale intensity” function was applied to rescale the intensity of the DAPI images by setting the brightest pixel in the picture to 1 and the darkest pixel to 0. This allowed for a reliable fixed thresholding detection of the nuclei in later analysis steps. The corrected images were exported into a new folder to be used in the subsequent pipeline.

#### Object detection and data acquisition

A second pipeline was designed for object recognition and data acquisition. First, corresponding images of both channels were read in and matched using the corresponding wavelength identifiers (w1=DAPI, w2=phalloidin). Metadata tags were extracted and stored in a database.

Next, DAPI stained nuclei were detected as primary objects. Too small objects as well as nuclei touching the border of the image were set to be eliminated from the analysis. The primary objects were then used to help detect the outlines of the cells in the phalloidin stained images. Hereby, the primary objects were set as starting points in the phalloidin image to propagate the signal towards the edge of the cell (area of highest intensity). In a third step of object recognition, the area of the cytoplasm was calculated by subtracting the primary object (nuclei area) from the secondary object (whole cell area). 

To exclude effects of cell-cell interactions on cell morphology, each cell was analyzed for the number of touching neighbors. Only fully separated cells (no contacts to neighboring cells) were kept for further analysis. All other cells were excluded. 

Finally, every object was analyzed for its phenotypic properties such as cell size (area), circularity or granularity. All measurements were written into an output excel file as well as an SQLite database. Images including outlines of separated cells were exported into a new folder and linked to the database. 

The time scale to run the two pipelines on one sub-experiment of 81 compounds was about 35 hours on an Intel Xeon E5-2687W workstation with 64GB of RAM. However, since the ***CellProfiler*** software package is currently only supporting a single core, analysis speed is similar in cheaper workstations with fewer cores. If speed however is an issue, analysis can be performed on several PCs, in a local cluster or - using the command line modus of ***CellProfiler*** – on a super computer cluster such as “Brutus” at the Swiss Federal Institute of Technology. 

#### Database access

To access the database and to retrieve and visualize the information generated from the assays, the software package ***CellProfilerAnalyst*** (www.cellprofiler.org) was used. First impressions of compound effects on cell morphology were obtained from non-normalized plate-specific heat maps of the selected values that have been stored in the database. Images of each plate position as well as the outlines of the analyzed cells were visualized via selecting the specific well in the heat maps. In a similar way thumbnail montages of all 24 images per well can be generated, allowing a quick and easy visual output of all images.

#### Phenotype classification using machine learning algorithms

For an unbiased phenotypic classification of spread vs. unspread cells, a machine-learning algorithm was implemented using the ***CellProfilerAnalyst*** software package. 100 cells were randomly fetched from each experiment, and manually sorted into either category: 1=spread or 2=unspread. Using machine learning algorithms, 20 rules were automatically generated, tested and weighed to automatically sort the cells by characteristics such as cell area, eccentricity or solidity. Classification accuracy was tested against the accuracy of a random classifier. Classification for one experiment required approximately 30 seconds of processing time. All classification data (spread cells, unspread cells and total cells) were stored in a database and exported into an excel-readable format. 

While ***CellProfilerAnalyst*** provided basic functionality to access and interpretate the data obtained, a more refined analysis was necessary to merge data, remove outliers, normalize data sets and select hits. For that a data handling pipeline (***supportingFileS3***) was constructed using the open source software package ***Knime*** (www.knime.org) with the following plugins/extensions: 

KNIME XLS Support 2.7.0.0035863 (org.knime.features.ext.poi.feature.group; KNIME GmbH, Konstanz, Germany); KNIME JFreeChart 2.7.0.0036050 (org.knime.features.ext.jfreechart.feature.group; KNIME GmbH, Konstanz, Germany); KNIME Itemset Mining 2.7.0.0035863 (org.knime.features.ext.itemset.feature.group; KNIME GmbH, Konstanz, Germany); KNIME HCS Tools 1.1.0.201209111420 (de.mpicbg.tds.knime.hcstools.feature.feature.group; Max Planck Institute of Molecular Cell Biology and Genetics (MPI-CBG))

#### Data merging and outlier removal

In a first step, data files from ***CellProfiler*** and ***CellProfilerAnalyst*** were imported and merged with spreadsheets containing different annotations (annotation files can be found in ***supporting*** File ***S3***). The data was grouped by its well identifiers and averaged. Plate viewer tools were installed throughout the pipeline to allow visualization of the data at each step. A scatter plot of the mean cell area per well enabled to identify and manually select outliers for removal.

#### Data normalization

Next, data was normalized in two steps. First, plate to plate variations were reduced using a percentage of control normalization (POC): values of each well were normalized to the corresponding average value of all control wells within the plate that did not contain any Nogo-A-∆20 coating. In a second step, a B-Scoring normalization (median averaging) was applied to correct for row and column effects. To allow B-score normalization of triplicates/ multiple substrates within one experimental plate, a row shuffling technique was developed. Rows without substrate were matched with corresponding rows from other plates. The same was done for substrate (Nogo-A-∆20) coated rows. After row shuffling, B-score normalizations were performed on these new virtual plate configurations that had the same coating for all wells and did not contain any replicates that would interfere with the B-Score normalization. The rows were then shuffled back to their original plate configurations and the results visualized in a heat map using a plate viewer tool.

#### Hit selection

A scatter plot module was implemented into the ***KNIME*** pipeline to allow for visualization of the normalized data. Potential hits were selected manually by highlighting and were exported into an excel readable format. To allow easy data plotting in statistical software such as Graph Pad Prism, a pivot table was generated and exported. Finally, also the complete list of all normalized values was exported as a raw list as well as a pivot table.

#### Final data presentation and statistics

Pivot tables containing the normalized data generated in ***KNIME*** were imported into ***GraphPadPrism6*** software (http://www.graphpad.com/scientific-software/prism/). Column graphs with standard errors of the mean (SEM) were plotted.

## Supporting Information

Figure S1 **Plate layout and pipetting scheme.** To allow for compound screening in the absence of expensive liquid handling robots, a pipetting scheme and plate layout was developed that reduces pipetting steps by using multi-channel pipettes. (**A**) *Grandmother plate (stock plate)*. Most compound libraries are delivered with a plate layout as depicted in (A). Each number refers to a specific compound while N refers to the negative control (e.g. DMSO). The NINDS library used followed this layout with 13 plates à 80 compounds (20μl, 1mM stocks in DMSO). (**B**) *Mother plates*. Using multi-channel pipettes, stocks are transferred in a row to row fashion (column to row for the blue labeled wells) into 384-well plates (“mother plates”; small volume reservoirs). Depending on the amount of positive controls required, controls are added to the mother plate. For 80 compounds to be tested in one experiment (9 final assay plates) we added one positive control (green well). Alternatively positive controls can be added for each plate. (**C**) *Baby plates*. Each row of the mother plate provides compounds for one final assay plate (“baby plate”). First, compounds are diluted in “daughter plates” (not shown). Then, they are transferred to baby plates. Every compound is being measured in triplicate on two different substrates (e.g. Nogo-A-Δ20 vs. control). If no substrate is to be used, the amount of tested compounds per plate can be doubled.
(TIF)

Figure S2 **Screening pipeline adaptation (e.g. for siRNA screens).** Spreading assay of 3T3 cells nucleofected with siRNA. Only cells positive for siGlo (nucleofection marker) are analyzed. Next to an image based analysis of the mean areas, object based analyses using binning as well as frequency distribution plots can be employed.: (**A/B**) *1° and 2° object recognition as described* in Figure ***3***. (A) Phalloidin channel; (B) Nuclei and cytoplasm outlines identified; (C/D) *Primary* *object* *recognition* *of* *nucleofection* *marker*. (C) SiGlow signals are detected as 1° objects following the same principles as for DAPI stained nuclei. (D) Outlines for detected siGlow objects (green). A size threshold is applied to exclude small punctuated background signals (arrow heads). (**E**) *Filtering of “separated” cells colocalizing with nucleofection marker*. A neighbor analysis (as depicted in Figure ***3***) combined with a relate-object function is being used to identify and filter out “separated” siGlo-positive cells (red outlines). (**F**-**H**) *Analysis of cytoplasm area*. The KNIME pipeline was modified to allow not only for an image based (F) but also an object based analysis of the mean areas (G/H). (G) Using a binning analysis module, all objects can be categorized in bins according to their cytoplasm areas. The amount of cells (in %) is plotted against the bins (10% intervals). (H) Object data can additionally be imported into statistical software such as ***GraphPadPrism 6*** to allow for plotting of cumulative frequency distributions. The relative frequency (in %) is plotted against the cumulated cytoplasm area (in μm^2^). - Magnification: Calibration bars (50µm) in *A* are also applicable to *B*-*E*.
(TIF)

Figure S3 **KNIME Pipeline for data normalization, hit selection and analysis.** An analysis pipeline was developed in ***KNIME*** to allow for data processing. The major steps in the pipeline are annotated and color-coded including steps such as data import, outlier removal, data normalization, visualization, hit selection, pivoting and data export. A high resolution version of this file can be found in ***supporting*** File ***S3***.
(TIF)

File S1 **ReNamer presets file.** Settings file to batch rename images and to incorporate meta tags.
(RNP)

File S2 **CellProfiler pipeline 1.** Pipeline as shown in Figure ***3*** (consisting of 2 parts: “Additional file 2A.cp” and “Additional file 2B.cp”) - to be imported into CellProfiler software package.
(ZIP)

File S3 **KNIME pipeline.** Pipeline as shown in Figure ***3*** - to be imported into KNIME software package. Zip folder includes a folder named “Additional file 3 which contains the “KNIME Pipeline – overview.pdf” (High resolution image of KNIME pipeline), the “KNIME Pipeline.zip” (Pipeline files for import into KNIME), the “Node 11 – Layout.xls” (Annotation file to be loaded into KNIME node 11), the “Shuffle Annotations.xls” (Annotation file for reshuffling).
(ZIP)

File S4 **CellProfiler pipeline 2.** Pipeline as shown in Figure ***2*** - to be imported into CellProfiler software package.
(CP) Click here for additional data file.
