# Supplementary figures and images for: An Open Source Based High Content Screening Method for Cell Biology Laboratories Investigating Cell Spreading and Adhesion
Source: PLoS One. 2013 Oct 21;8(10):e78212. doi: 10.1371/journal.pone.0078212 (PMC3804740; doi:10.1371/journal.pone.0078212)

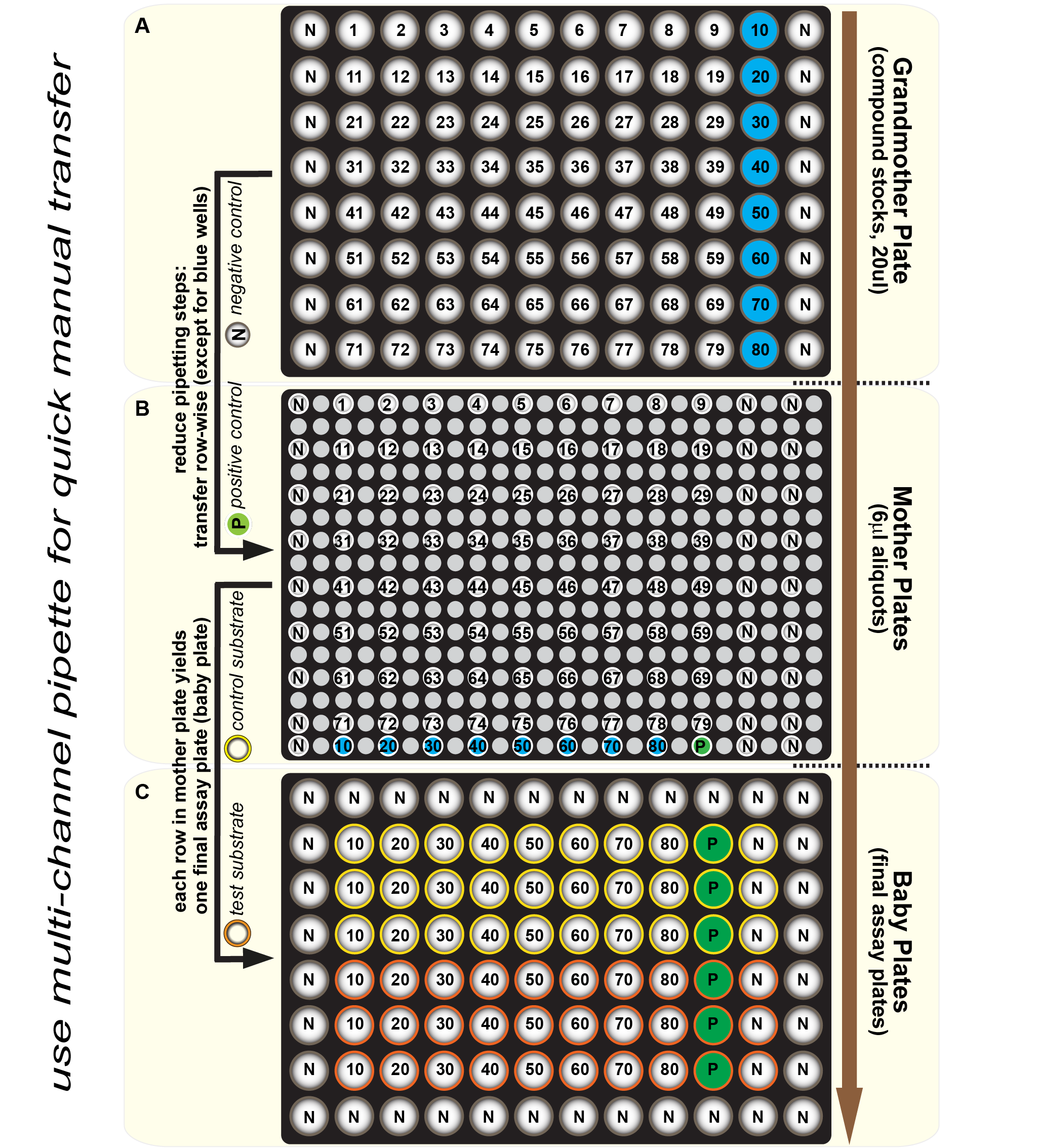

Supplement: Figure S1 — Plate layout and pipetting scheme. To allow for compound screening in the absence of expensive liquid handling robots, a pipetting scheme and plate layout was developed that reduces pipetting steps by using multi-channel pipettes. (A) Grandmother plate (stock plate). Most compound libraries are delivered with a plate layout as depicted in (A). Each number refers to a specific compound while N refers to the negative control (e.g. DMSO). The NINDS library used followed this layout with 13 plates à 80 compounds (20μl, 1mM stocks in DMSO). (B) Mother plates. Using multi-channel pipettes, stocks are transferred in a row to row fashion (column to row for the blue labeled wells) into 384-well plates (“mother plates”; small volume reservoirs). Depending on the amount of positive controls required, controls are added to the mother plate. For 80 compounds to be tested in one experiment (9 final assay plates) we added one positive control (green well). Alternatively positive controls can be added for each plate. (C) Baby plates. Each row of the mother plate provides compounds for one final assay plate (“baby plate”). First, compounds are diluted in “daughter plates” (not shown). Then, they are transferred to baby plates. Every compound is being measured in triplicate on two different substrates (e.g. Nogo-A-Δ20 vs. control). If no substrate is to be used, the amount of tested compounds per plate can be doubled. (TIF) [file pone.0078212.s001.tif]

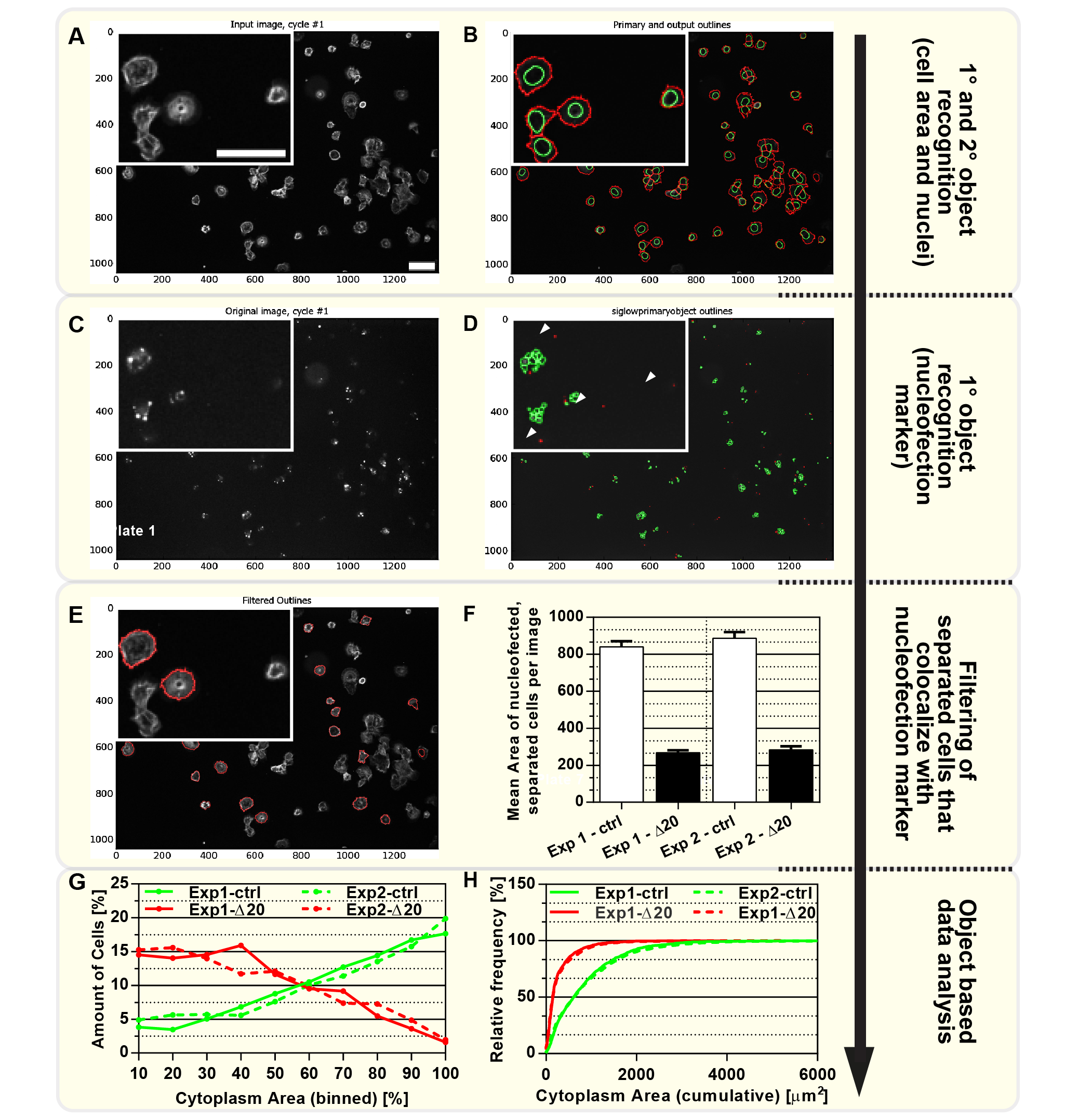

Supplement: Figure S2 — Screening pipeline adaptation (e.g. for siRNA screens). Spreading assay of 3T3 cells nucleofected with siRNA. Only cells positive for siGlo (nucleofection marker) are analyzed. Next to an image based analysis of the mean areas, object based analyses using binning as well as frequency distribution plots can be employed.: (A/B) 1° and 2° object recognition as described in Figure 3 . (A) Phalloidin channel; (B) Nuclei and cytoplasm outlines identified; (C/D) Primary object recognition of nucleofection marker. (C) SiGlow signals are detected as 1° objects following the same principles as for DAPI stained nuclei. (D) Outlines for detected siGlow objects (green). A size threshold is applied to exclude small punctuated background signals (arrow heads). (E) Filtering of “separated” cells colocalizing with nucleofection marker. A neighbor analysis (as depicted in Figure 3 ) combined with a relate-object function is being used to identify and filter out “separated” siGlo-positive cells (red outlines). (F-H) Analysis of cytoplasm area. The KNIME pipeline was modified to allow not only for an image based (F) but also an object based analysis of the mean areas (G/H). (G) Using a binning analysis module, all objects can be categorized in bins according to their cytoplasm areas. The amount of cells (in %) is plotted against the bins (10% intervals). (H) Object data can additionally be imported into statistical software such as GraphPadPrism 6 to allow for plotting of cumulative frequency distributions. The relative frequency (in %) is plotted against the cumulated cytoplasm area (in μm2). - Magnification: Calibration bars (50µm) in A are also applicable to B-E. (TIF) [file pone.0078212.s002.tif]

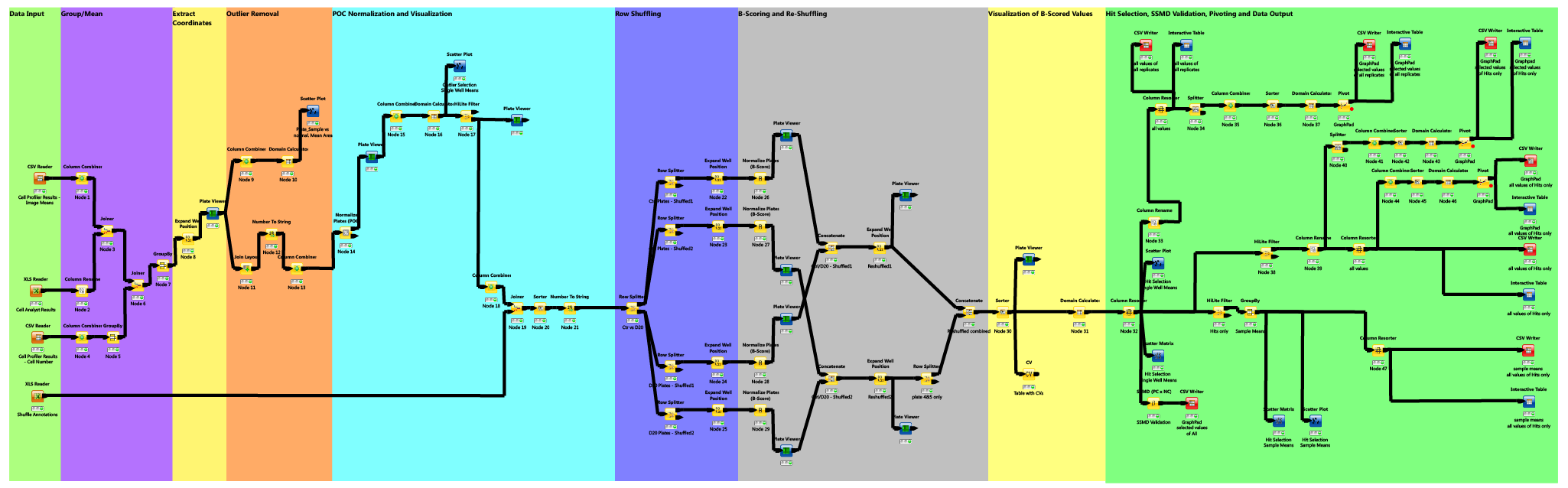

Supplement: Figure S3 — KNIME Pipeline for data normalization, hit selection and analysis. An analysis pipeline was developed in KNIME to allow for data processing. The major steps in the pipeline are annotated and color-coded including steps such as data import, outlier removal, data normalization, visualization, hit selection, pivoting and data export. A high resolution version of this file can be found in supporting File S3. (TIF) [file pone.0078212.s003.tif]

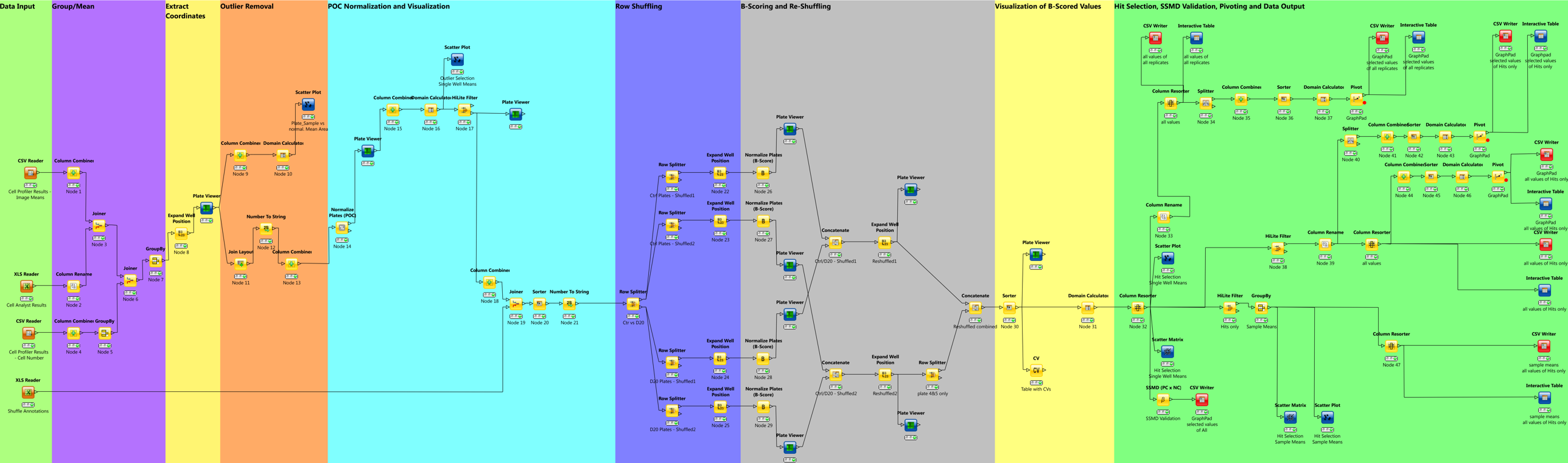

Supplement: File S3 — KNIME pipeline. Pipeline as shown in Figure 3 - to be imported into KNIME software package. Zip folder includes a folder named “Additional file 3 which contains the “KNIME Pipeline – overview.pdf” (High resolution image of KNIME pipeline), the “KNIME Pipeline.zip” (Pipeline files for import into KNIME), the “Node 11 – Layout.xls” (Annotation file to be loaded into KNIME node 11), the “Shuffle Annotations.xls” (Annotation file for reshuffling). (ZIP) [file pone.0078212.s006.zip › Additional file 3/KNIME Pipeline - overview.pdf]
